# Methylation at the C-3′ in D-Ring of Strigolactone Analogs Reduces Biological Activity in Root Parasitic Plants and Rice

**DOI:** 10.3389/fpls.2019.00353

**Published:** 2019-04-02

**Authors:** Muhammad Jamil, Boubacar A. Kountche, Imran Haider, Jian You Wang, Faisal Aldossary, Randa A. Zarban, Kun-Peng Jia, Djibril Yonli, Umar F. Shahul Hameed, Ikuo Takahashi, Tsuyoshi Ota, Stefan T. Arold, Tadao Asami, Salim Al-Babili

**Affiliations:** ^1^ The BioActives Lab, Division of Biological and Environmental Sciences and Engineering, King Abdullah University of Science and Technology, Thuwal, Saudi Arabia; ^2^ Institute of Environment and Agricultural Research (INERA), Ouagadougou, Burkina Faso; ^3^ Computational Bioscience Research Center, Division of Biological and Environmental Sciences and Engineering, King Abdullah University of Science and Technology, Thuwal, Saudi Arabia; ^4^ Graduate School of Agricultural and Life Sciences, The University of Tokyo, Tokyo, Japan

**Keywords:** strigolactones, *Striga hermonthica*, suicidal germination, shoot branching, strigolactone analogs

## Abstract

Strigolactones (SLs) regulate plant development and induce seed germination in obligate root parasitic weeds, e.g. *Striga* spp. Because organic synthesis of natural SLs is laborious, there is a large need for easy-to-synthesize and efficient analogs. Here, we investigated the effect of a structural modification of the D-ring, a conserved structural element in SLs. We synthesized and investigated the activity of two analogs, MP13 and MP26, which differ from previously published AR8 and AR36 only in the absence of methylation at C-3′. The de-methylated MP13 and MP26 were much more efficient in regulating plant development and inducing *Striga* seed germination, compared with AR8. Hydrolysis assays performed with purified *Striga* SL receptor and docking of AR8 and MP13 to the corresponding active site confirmed and explained the higher activity. Field trials performed in a naturally *Striga*-infested African farmer’s field unraveled MP13 as a promising candidate for combating *Striga* by inducing germination in host’s absence. Our findings demonstrate that methylation of the C-3′ in D-ring in SL analogs has a negative impact on their activity and identify MP13 and, particularly, MP26 as potent SL analogs with simple structures, which can be employed to control *Striga*, a major threat to global food security.

## Introduction

Strigolactones (SLs) are an intriguing class of plant hormones, which have attained immense importance during the past two decades because of their diverse biological roles in plant biology (Al-Babili and Bouwmeester, 2015; Waters et al., 2017). For example, SLs regulate several aspects of aboveground plant architecture, especially shoot branching/tillering and shoot secondary growth (Gomez-Roldan et al., 2008; Umehara et al., 2008; Scaffidi et al., 2014). SLs also determine the growth of different types of roots, i.e. primary, lateral, crown, and adventitious roots, as well as root hairs (Kapulnik et al., 2011; Ruyter-Spira et al., 2011; Sun et al., 2016). Furthermore, SLs regulate other processes, such as leaf senescence and root nodulation (Soto et al., 2010; Agusti et al., 2011; Foo and Davies, 2011; Yamada et al., 2014), and have been recently shown to be involved in pathogen defense and abiotic stress responses (Ha et al., 2014; Torres-Vera et al., 2014; Decker et al., 2017; Li et al., 2017; Lv et al., 2018; Zhang et al., 2018). Besides *in-planta* activities, SLs are released by plant roots into the rhizosphere as signaling molecules to establish beneficial symbiosis with arbuscular mycorrhizal (AM) fungi, by stimulating the branching of their hyphae (Akiyama et al., 2005; Akiyama and Hayashi, 2006; Besserer et al., 2006). However, this signal is also perceived by seeds of root parasitic weeds of the *Orobanchaceae* family, which abuse SLs as germination signal, indicating the presence of a host that is needed for their survival as obligate parasites (Cook et al., 1966).

SLs comprise a group of carotenoid-derived, secondary metabolites with specific structural features. There are around 25 natural SLs with elucidated structure characterized by butenolide lactone ring, called D-ring, which is linked in *R* configuration to a second moiety that consists of a tricyclic lactone (ABC-ring) in canonical SLs, such as strigol and orobanchol. Non-canonical SLs, such as carlactonoic acid, contain less conserved second moiety instead of the ABC-ring (Al-Babili and Bouwmeester, 2015; Jia et al., 2018). SLs are synthesized through a pathway that involves a 9-*cis*/all-*trans*-β-carotene isomerase DWARF27 (D27) and CAROTENOID CLEAVAGE DIOXYGENASE 7 (CCD7) and CCD8. These three enzymes are plastid-localized and conduct the steps converting all-*trans*-β-carotene into the key intermediate carlactone (CL) (Alder et al., 2012; Bruno et al., 2016, 2017; Bruno and Al-Babili, 2016; Abuauf et al., 2018). CL is then exported into the cytosol where cytochrome P450 (711 clade; homologs of the *Arabidopsis thaliana*) MORE AUXILLARY GROWTH 1 (MAX1) homologs and other enzymes convert it into SLs (Abe et al., 2014; Zhang et al., 2014; Al-Babili and Bouwmeester, 2015; Brewer et al., 2016). Plant SL receptors such as the rice D14 belong to the α/β hydrolases that are characterized by a conserved Ser-His-Asp catalytic triad and which bind and hydrolyze the SL ligand into the characteristic D-ring and the corresponding second moiety (Hamiaux et al., 2012; de Saint Germain et al., 2016; Yao et al., 2016; Shabek et al., 2018). Following hydrolysis, D14 covalently binds the released D-ring and undergoes structural rearrangements, which is recognized by the downstream component MAX2 (Jia et al., 2016; Waters et al., 2017). KARRIKIN INSENSITIVE 2 (KAI2) proteins are D14 homologs that bind smoke-derived chemicals karrikins instead of SLs (Waters et al., 2012a). It is assumed that karrikins mimic a yet unidentified plant hormone/signaling molecule. Seeds of root parasitic plants germinate only after perceiving host-released SLs by a set of receptors *Striga hermonthica* HYPOSENSITIVE TO LIGHT (ShHTLs), which have evolved through extensive gene duplication of KAI2/HTL in *Orobanchaceae* and new functionalization that enables sensing SLs (Conn et al., 2015; Toh et al., 2015). ShHTL7 is by far the most sensitive *Striga* SL receptor, indicating its crucial role in the germination process (Yao et al., 2017). This presumed role has been very recently confirmed by demonstrating the effect of ShHTL7 inhibitor(s) on *Striga* seed germination (Holbrook-Smith et al., 2016; Mashita et al., 2016; Hameed et al., 2018; Hamiaux et al., 2018; Nakamura et al., 2018).

Root parasitic weeds, especially *Striga* spp. (Witchweeds), and *Phelipanche* and *Orobanche* spp. (Broomrapes), are of great economic importance, as they infest a wide range of cereal and non-cereal crops, causing enormous yield losses (Ejeta, 2007; Parker, 2009; Mandumbu et al., 2019). These parasites have developed a host recognition strategy, by sensing host-released underground signaling molecules, particularly SLs. After germination, emerging parasites develop a haustorium that connects to the vascular system of the host, to uptake water, minerals, and photosynthetic products (Xie et al., 2010). Most of the damage occurs at this underground phase. Although a number of manual, cultural, and mechanical control measures have been adopted by the farmers, the infestation of these parasites is spreading over large areas due to enormous production of thousands of long-lasting tiny seeds. Indeed, root parasitic weeds have become a severe agricultural problem. In particular, *S*. *hermonthica*, which devastates cereal yields, has now been considered as one of the most serious biological threats to global food security (Pennisi, 2010). A further example is *Phelipanche* spp. that attack non-cereal crops in Eastern Europe and Asia (Parker, 2009, 2012). One promising strategy to alleviate the infestation of these parasites is to reduce the seed banks accumulated in infested soils. The SL dependency of parasitic seeds for germination can be exploited to clean off heavy infested soil by pursuing the so-called “suicidal” approach (Zwanenburg et al., 2016a). This approach refers to the application of germination stimulants, e.g. SLs or SL analogs, in the absence of a host, which leads to the death of arising parasite seedlings within short time (Kgosi et al., 2012).

Due to difficulties and large cost of organic synthesis of natural SLs (Zwanenburg et al., 2009; Bromhead and McErlean, 2017; Yasui et al., 2017), there is a large demand for analogs that are required to decipher SL functions in basic research as well as for applications, such as combating root parasitic weeds by suicidal germination. Indeed, many synthetic SL analogs have been designed and synthesized to test the function of SL in various biological processes, and some of them have been already tested as suicidal germination agents (Sugimoto et al., 1998; Zwanenburg et al., 2016a; Jamil et al., 2018). However, production of many synthetic analogs is also difficult because of a laborious and multistep synthesis process. Hence, designing of simple and cheaper SL analogs with reasonable efficiency is very important to allow their large-scale usage (Zwanenburg et al., 2009; Boyer et al., 2014). Several efforts have been made in the past to generate simple SL analogs. The most commonly used standard SL analog GR24 was systematically simplified by removing A-ring (GR7) or by eliminating B-ring (GR5). Interestingly, all three compounds stimulate parasitic seed germination (Zwanenburg et al., 2016b). This information further led to the modification of SL analogs into simple forms that require few synthesis steps. For example, Nijmegen-1, a simple SL analog, was developed and showed high activity in inducing parasitic seed germination (Zwanenburg et al., 2016c). SL analogs were also modified by isosteric replacement of a specific atom like imino SL analogs (Kondo et al., 2007) and strigolactams (Lachia et al., 2015). There are also SL mimics that contain only D-ring attached with an α, β-unsaturated carbonyl and an enol ether bridge but without ABC scaffold. Debranones and arolyloxy SL mimics showed inhibition of shoot branching (Fukui et al., 2011, 2013) and *Striga* seed germination. Based on the structure and biological activity of the intermediate carlactone (Alder et al., 2012) and its derivative methyl carlactonoate (Abe et al., 2014), we have recently developed the simple SL analogs nitro-phenlactone and methyl-phenlactonoates (MPs) (Jia et al., 2016; Jamil et al., 2018). These analogs, in particular some MPs, showed high efficiency in exerting specific SL functions (Jamil et al., 2018).

Boyer et al. (2014) reported on structurally simple series of SL analogs (ARs), including AR8 and AR36 that comprise only a D-ring with an extra methyl group at the C-3′ position (dimethyl butenolide motif) along with an enol ether bridge and an acyclic carbon chain. Although the AR series is very simple and easy to synthesize, they showed weak activities in rhizosphere, particularly in inducing seed germination of root parasitic weeds (Boyer et al., 2014). Since SLs have been reported to regulate specific activity in a structure-dependent manner (Zwanenburg and Pospisil, 2013; Artuso et al., 2015; Dvořáková and Vaněk, 2015), we proposed that modification of ARs’ structure might increase their efficiency in exerting specific SL functions. Here, we developed the SL analogs MP13 and MP26 by removing the C-3′ methyl group of AR8 and AR36, respectively. Activity tests demonstrated the high positive impact of this modification on the activities of SL analogs.

## Materials and Methods

### Plant Materials and Growth Conditions

Seeds of *S. hermonthica* were collected from Sorghum field near Wad Medani, Sudan (courtesy of Prof. A.G. Babiker). Seeds of *P. aegyptiaca* (collected from tomato field) were provided by Prof. Mohamed Ewis Abdelaziz, Cairo University, Egypt. Seeds of rice *cv* IAC 165 were obtained from Africa Rice, Tanzania (courtesy of Jonne Rodenburg). The seeds of *d10* and *d3* mutants were kindly provided by Dr Junko Kyozuka, Tohoku University, Japan. *Arabidopsis* (Columbia) seeds were multiplied locally. Rice was grown at 28°C and 70% relative humidity with fluorescent white light (130–180 μM m^−2^ s^−1^) 12-h day/night period. For root and hypocotyl studies, *Arabidopsis* plants were grown 16 h at 22°C/8 h at 16°C day/night, 60% relative humidity, and 60–70 μmol m^−2^ s^−1^ white light. *Striga* preconditioning and germination were carried out at 30°C under moist conditions in the dark.

### Chemicals and Formulation

The SL analog AR8 was synthesized according to Boyer et al. (2014). The emulsifier Atlas G1086 (Croda, Netherlands) and the standard SL analog GR24 were kindly provided by Prof. Binne Zwanenburg, Radboud University, Netherlands. GR24 was applied as a racemic mixture (*rac*-GR24). Cyclohexanone was purchased from Sigma-Aldrich. The formulated emulsifier was prepared as previously described (Zwanenburg et al., 2016a). About 5 ml of Atlas G 1086 was added in 20 ml of cyclohexanone and agitated well. Then respective amount of this emulsifier was added to each SL analog to prepare 10 mM stock solution. A number of other chemicals used to prepare half-strength Hoagland’s nutrient solutions, 2-(N-morpholino)ethanesulfonic acid (MES), and dimethyl sulfoxide (DMSO) buffer were purchased from different suppliers.

### Synthesis of (*E*)-2-methyl-3-[(4-methyl-5-oxo-2,5-dihydrofuran-2-yl)oxy] acrylaldehyde (MP13)

3-Dimethylamino-2-methyl-2-propenal (272 mg, 2.4 mmol, purchased from market) and 7.7 N NaOH aq 390 μl (1.1 eq, 3.0 mmol) were heated at 70°C until the reaction mixture became homogeneous. Then the reaction mixture was dried with evaporation under reduced pressure. To the solution of the resultant residue in DMSO (5.0 ml), 5-bromo-3-methyl-2(5*H*)-furanone (725 mg, 4.1 mmol) (Wolff and Hoffmann, 1988) was added slowly and stirred overnight. The reaction mixture was diluted with ethyl acetate and the organic layer was washed successively with water and saturated sodium chloride solution, dried (Na_2_SO_4_), and evaporated. The residue was column chromatographed on silica gel using a mixture of hexane and ethyl acetate as eluent to give (E)-2-methyl-3-[(4-methyl-5-oxo-2,5-dihydrofuran-2-yl)oxy]acrylaldehyde (MP13). Yield was 57%. The physicochemical properties of MP13 are: ^1^H NMR (500 MHz, CDCl_3_): δ 9.34 (1H, s), 7.17 (1H, s), 6.98 (1H, s), 6.19 (1H, s), 2.04 (3H, s), 1.70 (3H, s). HRMS-Esi: m/z [M-Na]^−^ Calcd for C_9_H_10_Na_1_O_4_:205.04768, found: 205.06819. 13C-NMR (126 MHz, CDCl3) δ: 192.19, 170.54, 162.58, 141.40, 135.18, 122.52, 100.29, 10.38, 6.20. Melting point was 88°C (Table S2).

### Synthesis of (2*E*,4*E*)-methyl 4-methyl-5-(4-methyl-5-oxo-2,5-dihydrofuran-2-yloxy)penta-2,4-dienoate (MP26)

Methyl (triphenylphosphoranylidene) acetate (2.1 mmol) was mixed with MP13 (1.13 mmol) and toluene (20 ml/mmol) in a round-bottom flask and stirred for 23 h at room temperature. Then, the reaction mixture was concentrated *in vacuo* with an evaporator to give residue, which was then purified with silica gel column with ethyl acetate-hexane as eluent to give MP26 in 55% yield. The physicochemical properties of MP26 are: ^1^H NMR (500 MHz, CDCl_3_): δ 7.25 (1H, d, *J* = 15 Hz), 6.91 (1H, s), 6.73 (1H, s), 6.06 (1H, s), 5.81(1H, d, *J* = 15 Hz), 3.74 (3H, s), 2.00 (3H, s), 1.74 (3H, s). 13C-NMR (126 MHz, CDCl3) δ: 170.70, 167.66, 148.56, 144.82, 141.55, 135.22, 117.16, 115.06, 100.00, 51.39, 10.62, 9.31. Melting point was 146–149°C (Table S2).

### Rice Micro Tillering Bioassays

Rice seeds (*d10*) were sterilized with 2.5% sodium hypochlorite for 15 min. After thorough washing with sterile MilliQ water, the seeds were kept in water for 2 days at 30°C in the dark for imbibition and pre-germination. The pre-germinated seeds were then transferred to moist filter paper in 90-mm petri plates and incubated at 30°C overnight. The plates with germinated seeds were then shifted in a growth cabinet with fluorescent white light (130–180 μM m^−2^ s^−1^) for 1 week. Seven days-old seedlings were moved to 50-ml falcon tubes (one seedling per tube) containing modified half-strength Hoagland nutrient solution in greenhouse. After 1 week, rice plants were treated with 2.5 μM of each SL analog. Mock and GR24 (2.5 μM) were included as control treatments. Six applications of each SL analog were done two times in a week. After 3 weeks of application, number of tillers per plant, plant height, and fresh biomass were measured at final harvest.

### *Arabidopsis* Lateral Root Density and Hypocotyl Elongation Assays

Half-strength MS prepared with 0.5 gl^−1^ MES along with 0.5% sucrose and 1% agar at pH 5.7 were added in petri plates supplemented with SL analogs (2.5 μM). The sterilized *Arabidopsis* seeds were grown in petri plates that were first kept at 4°C in darkness for 3 days. For hypocotyl study, the petri plates were exposed to continuous white light for 24 h, then transferred to continuous monochromatic red light (20 μmol m^−2^ s^−1^, 22°C) conditions for another 4 days. For lateral root density, the plates were vertically grown at 22°C in Percival incubator under long day conditions (16 h at 22°C/8 h at 16°C day/night, 60% relative humidity, 60–70 μmol m^−2^ s^−1^ white light) for 8 days. After scanning, the hypocotyl length and number of lateral roots were recorded using publicly available ImageJ software.^1^

### Parasitic Seed Germination Bioassays

Germination activity of SL analogs on *S. hermonthica* and *P. aegyptiaca* seeds was tested by following the procedure described before (Jamil et al., 2012). The preconditioned *Striga* and *Phelipanche* seeds were treated with 50 μl of each SL analog solution at a concentration range (10^−5^–10^−12^ M). GR24 and sterile MilliQ water were included as positive and negative controls, respectively. The treated seeds were allowed to germinate in the dark for 24 h at 30°C (for *Striga*) and for 1 week at 25°C (for *Phelipanche*). The germinated and non-germinated seeds were counted under a binocular microscope, and germination rate (in %) was calculated.

### Stability of SL Analogs

The SL analogs GR24, AR8, MP13, and MP26 were first tested for their stability by high-performance liquid chromatography (HPLC) in an aqueous solution (50 μgml^−1^, pH, 6.8) by adopting the previously described procedure with minor modifications (Jamil et al., 2018). Each compound (1 mg) was dissolved in 1-ml acetone, and then 75 μl out of the analog solution were diluted with 150 μl ethanol and 750 μl water. Thereafter, 25 μl Indanol (1 mg ml^−1^, internal standard) was spiked in 975-μl test solution. The time course of degradation was monitored by ultra-performance liquid chromatography (UPLC) analysis using a Zorbax Eclipse XDB-C18 column (3.5 μm, 4.6 mm× 150 mm), eluted first by 5% acetonitrile in water for 0.5 min, then by a gradient flow from 5 to 100% acetonitrile within 17.5 min, and by 100% acetonitrile for 5 min. The column was operated at 40°C with a flow rate of 0.35 ml min^−1^, and the temperature of auto-sampler was set at 21°C. Compounds eluted from the column were detected with a photodiode array detector, and the relative quantity of non-degraded amount was calculated by integrating corresponding peak areas and comparison with the internal standard Indanol. Stability was monitored at 3-day intervals up to 3 weeks.

The stability of formulated SL analogs was further tested in sand culture. About 1.5 ml of silver sand was added in each of 6 wells of 24-well plates. Then, 800 μl of formulated SL analogs (1.0 μM) was added in each of the six wells. The plates were sealed firmly with para-film, wrapped in aluminum foil, and placed at 30°C from day 1 to day 7. After 7 days, glass fiber discs containing preconditioned *Striga* seeds were placed on the top of each of the six wells and incubated in the dark at 30°C for 24 h. The germinated and non-germinated seeds were counted from each disc. In addition, the viability of formulated SL analogs was also tested in aqueous solution for 7 weeks at 30°C. About 800-μl aqueous solution of formulated SL analogs (1.0 μM) was added in a black Eppendorf tube weekly up to 6 weeks and incubated at 30°C. On the seventh week, about 50 μl of each analog was applied on a glass fiber disc containing preconditioned *Striga* seed and put again in the dark in sealed petri plates at 30°C for 24 h. Germinated and non-germinated seeds were counted from each disc.

### *In vitro* Yoshimulactone Green Assays

Protein expression and purification of ShHTL7 and *Oryza sativa* D14 (OsD14) was carried out according to the procedure described earlier (Jamil et al., 2018). *In vitro* Yoshimulactone Green (YLG) hydrolysis assays (Tsuchiya et al., 2015) were carried out using 3.0 μM of protein ShHTL7 and OsD14 in a reaction buffer (1X PBS buffer, pH 7.3) with 0.1% DMSO at a 100-μl volume on a 96-well black plate (Greiner). For competitive assays, *rac*-GR24, AR8, MP13, and MP26 (at range between 0.01 and 50 μM) were co-incubated with 1.0 μM of YLG (Tokyo Chemical Industry Co. Ltd., product number E1238) for 60 min at room temperature. Fluorescent intensity was measured by spectraMax i5 (Molecular Devices) at excitation by 480 nm and detection by 520 nm. The change in fluorescence observed over the course of 1 h of YLG in buffer without protein was subtracted from the data collected in presence of protein. IC_50_ values were calculated by GraphPad (Prism 7).

### Docking of AR8 and MP13 to ShHTL7 and OsD14 Active Site

AR8 and MP13 were docked to ShHTL7 (pdb:5z8p) and OsD14 (pdb:5dj5) based on the structure bound to GR24 (pdb:5dj5) as template using COOT (Emsley and Cowtan, 2004) program. The resulting pdb files were used for making final pictures using the PYMOL program. The amino acids of ShHTL7 and OsD14 forming close contacts with AR8 and MP13 based on the docking are identified using Ligplot Program (Wallace et al., 1995).

### *Striga* Emergence in Pots Under Greenhouse Conditions

About 10 mg of (*ca* 4,000) *Striga* seeds were thoroughly mixed in 2 L of sand and soil mixture (1:1) and added in 3-L perforated plastic pot containing 0.5 L of clean soil in the bottom. *Striga* seeds were first preconditioned by giving light irrigation and putting pots at 35°C in greenhouse for 10 days. Each pot was sprayed with 20-ml (1.0 μM) formulated SL analogs up to 10 days at 2-day intervals. A light irrigation was given next day of each application to move the compound to *Striga* seeds and they were allowed to germinate for suicidal death without host for another 1 week. Then, three rice seedlings (5 days old) were planted in the middle of each pot. The rice plants were grown under normal growth condition (30°C, 70% RH) for 8 weeks. Number of emerged *Striga* plants in each pot were counted.

### *Striga* Emergence in Response to Suicidal Germination of Formulated MP13 Application in Naturally Infested Farmer’s Field

A naturally *Striga*-infested farmer’s field was selected near Kouaré research station (11°95′N, 0°30′W) (Fada N’Gourma, Burkina Faso) to test suicidal activity of formulated MP13, taking formulated water treatment as control treatment. After onset of few rains, the field was thoroughly prepared by subsequent ploughing and planking. The experiment was laid out in a randomized complete block design with four plots or replications. Each plot consisted of two 3-m-long mounds and spaced with one mound between rows. Formulated MP13 and normal water (control) were applied twice in each plot (25 ml m^−2^) following rains (~10 mm) to get a final concentration of 0.5 μM. Local pearl millet crop was sown at least 1 week after the second application. Data on the number of emerged *Striga* plants at three counting dates were collected in both fields on a plot basis. The three *Striga* counts were then used to calculate the average number of emerged *Striga* plants throughout the season.

### Statistical Analyses

Collected data were analyzed statistically using statistical software package R (version 3.2.2). One-way analysis of variance (ANOVA) and least significant difference (LSD) multiple range test were used for analyzing the effect of different SL analogs on *Striga* infestation and plant biology. Half maximum effective concentration (EC_50_) was calculated using IC_50_ toolkit.^2^ Synthetic strigolactone analog GR24 and AR8 were used as standards for comparison with MP13 and MP26.

## Results

### Synthesis and Physicochemical Properties of MP13 and MP26

**Figure 1:**
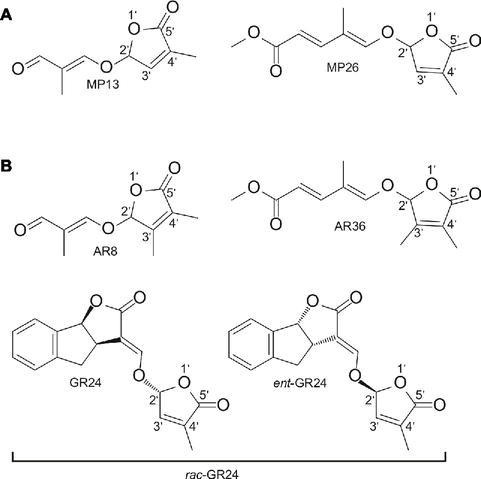
**(A)** Structure of modified simple SL analogs MP13, MP26; **(B)** Structure of parent SL analogs AR8, AR36, and standard SL analog *rac*-GR24 consisting of the two shown stereoisomers. MP13 and MP26 were developed by structural modification from previously described SL analogs AR8 and AR36, respectively (Boyer et al., 2014).

**Figure 2:**
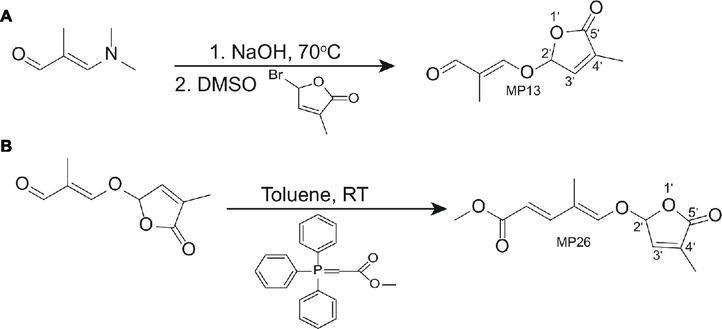
**(A)** Synthesis of (E)-2-methyl-3-[(4-methyl-5-oxo-2,5-dihydrofuran-2-yl)oxy]acrylaldehyde (MP13). **(B)** Synthesis of (2E,4E)-methyl 4-methyl-5-(4-methyl-5-oxo-2,5-dihydrofuran-2-yloxy)penta-2,4-dienoate (E)-methyl 3-(4-methyl-5-oxo-2,5-dihydrofuran-2-yloxy)-2-(4-nitrophenyl)acrylate (MP26).

The two SL analogs MP13 and MP26 differ from the previously described AR8 and AR36 (Boyer et al., 2014) by the absence of the methyl group at the C-3′ in the D-ring. The structures of the four compounds are shown in Figure 1. MP13 and MP26 were synthesized following the procedure depicted in Figure 2.

### MP13 and MP26 Show Higher Activity Than AR8 in Regulating *d10* Rice Tillering and *Arabidopsis* Hypocotyl/Root Growth

**Figure 3:**
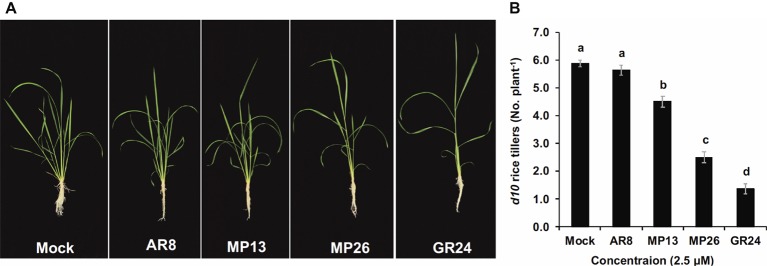
**(A)** Tillering phenotype of *d10* mutant in response to SL analogs’ application. **(B)** Effect of SL analogs on tillering inhibition. The SL analogs were applied (2.5 μM) to 1-week old hydroponically grown rice seedlings (*d10* mutant) twice a week up to 3 weeks. Number of tillers per plant were recorded (*n* = 8) and compared by one-way ANOVA. Means not sharing a letter in common differ significantly at *p*_0.05_.

First, we examined the potential of MP13 and MP26 in exerting SL growth-regulating activities. In one study, we tested their capability in rescuing the high-tillering phenotype of the SL-deficient *d10* mutant grown hydroponically. On an average, the high-tillering *d10* mutant showed six tillers per plant. This number decreased to 4.5 (23% inhibition) and 2.5 tillers per plant (57% inhibition), upon treatment with MP13 and MP26, respectively. Treatment with GR24 reduced the number of tillers to 1.4 tillers per plant (76% inhibition) while AR8 showed the weakest effect (5.6 tillers per plant) (Figures 3A,B). Application of GR24 and MP26 also showed significant increase in plant height as compared to mock with dwarf rice plant while rice biomass remained unaffected by all SL analogs (Figure S1). In another study, we studied the effect of MP13 and MP26 on *Arabidopsis* shoot and root growth (Figure S2). Seedlings treated with MP26 (1.0 μM) showed significant inhibition of hypocotyl growth (15%) and lateral root density (43%), compared to control seedlings. In contrast, application of MP13 and AR8 did not lead to a significant effect. The standard SL analog GR24 showed again highest activity, causing a 37 and 53% reduction in hypocotyl length and lateral root density, respectively.

### Parasitic Seed Germination in Response to SL Analogs Under Lab Conditions

**Figure 4:**
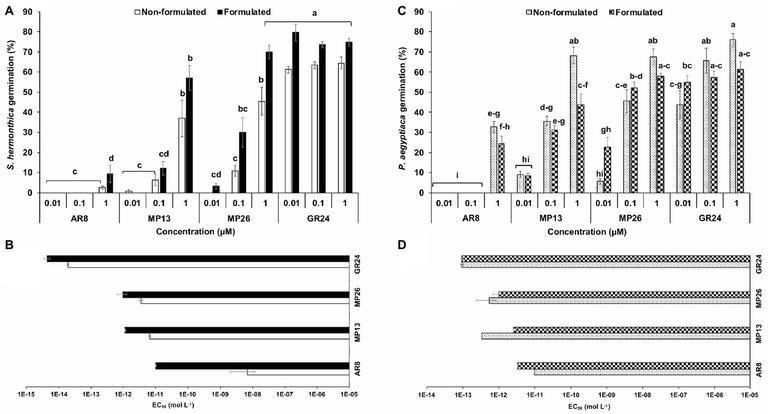
**(A,C)** *Striga hermonthica* and *Phelipanche aegyptiaca* seed germination-inducing activity. **(B,D)** Half maximal effective concentration (EC_50_) in response to various doses of formulated and non-formulated AR8, MP13, MP26, and GR24 treatments in petri plates. Preconditioned *S. hermonthica* and *P. aegyptiaca* seeds were treated with 50 μl each of aqueous solutions of AR8, MP13, MP26, and GR24 at 10^−5^–10^−12^ M. Bars represent percentage germination (*n* = 6), compared by one-way ANOVA and LSD_0.05_. Means not sharing a letter in common differ significantly at *p*_0.05_.

Firstly, new SL analogs were applied on preconditioned *Striga* seeds to examine their germination-inducing activity (Figure 4A). Application of both formulated and non-formulated MP13 and MP26 at 1.0 μM concentration led to very high *Striga* germination rate (37–70%), compared to the parent analog AR8 (9%). However, the activity of MP13 and MP26 was slightly lower than that of the standard SL analog GR24 (75%). To further validate these results, related half maximal effective concentration (EC_50_) of formulated and non-formulated SL analogs was determined (Figure 4B). Both formulated and non-formulated GR24 showed the highest activity with EC_50_ values of 4.4 × 10^−10^ (formulated) and 1.9 × 10^−9^ M (non-formulated). MP26 was the second most effective analog, with EC_50_ values of 9.8 × 10^−8^ (formulated) and 3.5 × 10^−7^ (non-formulated), followed by MP13, with EC_50_ values of 1.1 × 10^−7^ (formulated) and 6.4 × 10^−7^ M (non-formulated). AR8 exhibited the lowest activity and the highest EC_50_ value for formulated (9.9 × 10^−7^ M) and non-formulated (6.9 × 10^−4^ M) application.

We also applied MP13 and MP26 to preconditioned *P. aegyptiaca* seeds and determined their germination-inducing activity in this root parasitic plant. Application of non-formulated MP13 and MP26 at 1.0 μM concentration led to germination rate of around 68%, which is much higher than that of AR8 (32%) and similar (statistically equal) to that of GR24 (75%) (Figure 4C). We also calculated the EC_50_ values of formulated and non-formulated analogs. Here again, GR24 exhibited the highest activity with the lowest EC_50_ value (9.3 × 10^−9^ M, formulated), followed by MP26 (9.9 × 10^−8^, formulated; 5.2 × 10^−7^, non-formulated). Surprisingly, non-formulated MP13 showed better performance with less EC_50_ value (3.2 × 10^−8^), compared with formulated application (2.5 × 10^−7^ M). The previously published analog AR8 showed again highest EC_50_ values in both formulated (3.2 × 10^−7^ M) and non-formulated (9.7 × 10^−7^ M) application (Figure 4D).

### Stability of SL Analogs

**Figure 5:**
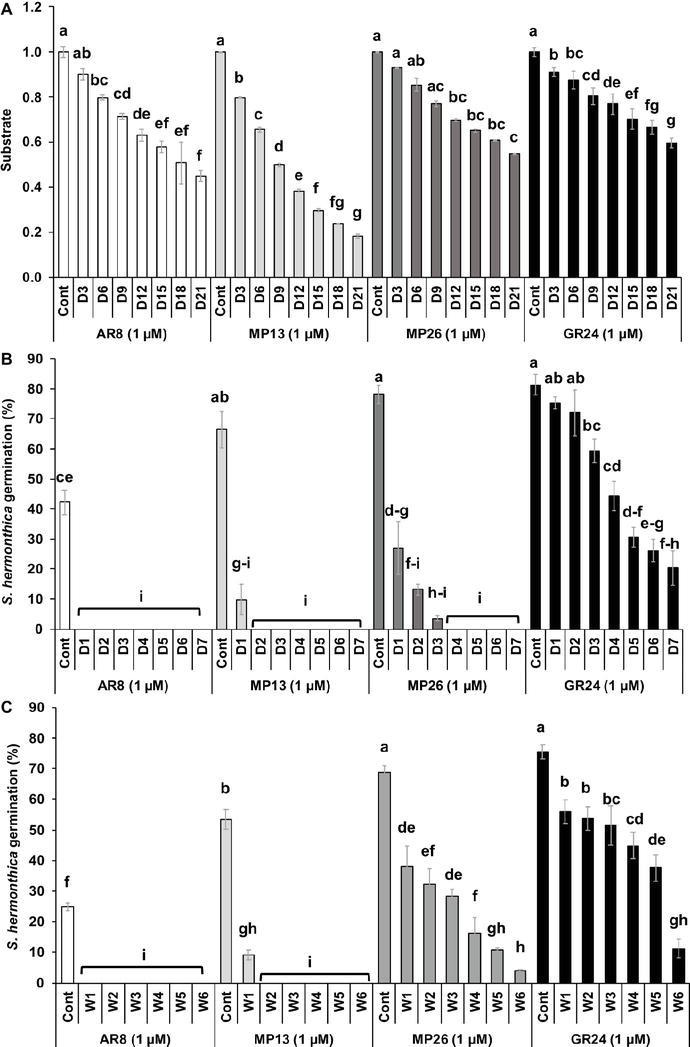
**(A)** Stability of SL analogs by HPLC analysis. The relative amount of non-degraded analogs was monitored in HPLC at 3 days’ interval up to 3 weeks and determined by comparison with internal standard. Data are means ± SE (*n* = 3). X-axis: time (days); Y-axis: substrate. **(B)** Stability of formulated SL analogs in sand. The formulated SL analogs were incubated at 30°C in sand daily up to 7 days. *Striga* germination was counted from each disc treated with SL analogs from respective days. **(C)** Stability of formulated SL analogs in aqueous solution. The formulated SL analogs were incubated at 30°C in aqueous solution weekly up to 6 weeks. *Striga* germination was counted from each disc treated with SL analogs from respective weeks. Data are means ± SE (*n* = 3) and compared by one-way ANOVA. Means not sharing a letter in common differ significantly at *p*_0.05_.

The stability of MP13 and MP26 was compared to the parent SL analogs AR8 and GR24 (Figure 5A). Initially, the modified analog MP26 displayed equal stability as GR24 up to 6 days of incubation and afterward it became less stable than GR24 but remained more stable than AR8. After 3 days, AR8 showed slightly less stability than GR24 and MP26 but more than MP13. MP13 exhibited the lowest stability throughout the whole 3 weeks of analysis.

The stability of MP13 and MP26 was also studied in sand culture at 30°C for 7 days (Figure 5B). Incubation of SL analogs for 1 day in sand led to a decrease in the seed germinating activity of MP13 and MP26 from around 60% to 10 and 27%, respectively. Two days of incubation of SL analogs resulted in a complete loss of the activity of MP13, while the activity of MP26 continued to decrease gradually till day 3 and was not detectable after this time point. GR24 showed a gradual decrease in activity over the whole incubation time and showed a germination rate of around 20% on seventh day of incubation. In contrast, AR8 completely lost its ability to induce *Striga* germination after 1 day of incubation in sand.

We also tested the stability of formulated SL analogs under prolonged storage (up to 6 weeks at 30°C) in aqueous solution, using *Striga* seed germinating activity (germination rate) as a readout (Figure 5C). The activity of MP13 decreased after 1 week’s storage from 53 to 9% and was not detectable after 2 weeks of storage. MP26 showed a continuous loss of activity from 69% before storage down to 4% after storage for 6 weeks. GR24 was more stable throughout the storage period and showed a decrease from 76 to 11% after 6 weeks of incubation. Remarkably, GR24 maintained an activity of around 38% for 5 weeks. AR8 showed a total loss of activity after 1 week’s storage.

### YLG Hydrolysis and Docking to ShHTL7 and OsD14

**Figure 6:**
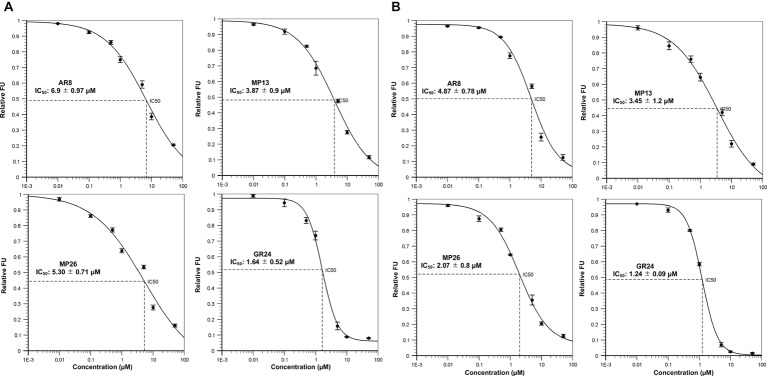
*In vitro* YLG (Yoshimulactone Green) assays. Competitive inhibition of **(A)** ShHTL7-mediated and **(B)** OsD14-mediated YLG hydrolysis by SL analogs. Seven concentrations ranging from 0.01, 0.1, 0.5, 1.0, 10, and 50 μM were applied and IC_50_ values for SL analogs were calculated.

**Figure 7:**
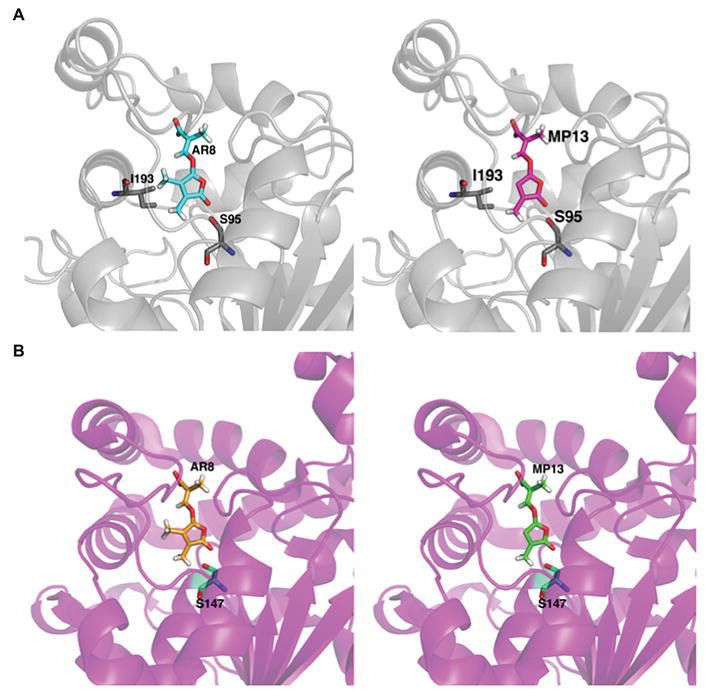
*In silico* structural analysis of MP13 and AR8 binding toward ShHTL7 and OsD14. **(A)** Cartoon representation of ShHTL7 (pdb: 5Z8P, gray) bound to AR8 (blue stick) and MP13 (magenta stick) and **(B)** OsD14 (pdb: 5dj5, magenta) bound to AR8 (Orange stick) and MP13 (green stick).

Next, we tested the interaction of MP13 and MP26 with the most sensitive *Striga* SL receptor in ShHTL7 (*Striga hermonthica Hyposensitive to Light 7*) (Toh et al., 2015) and rice SL receptor in OsD14. For this purpose, we determined the capability of these analogs in competing with YLG in ShHTL7- and OsD14-mediated hydrolysis assays, in comparison with AR8 and GR24 (Figures 6A,B). Results obtained unraveled highest ShHTL7 binding activity for GR24 (IC_50_: 1.64 ± 0.52 μM), followed by MP13 (IC_50_: 3.87 ± 0.90 μM), MP26 (IC_50_: 5.30 ± 0.71 μM), and finally AR8 (IC_50_ of 6.90 ± 0.97 μM). Similarly, highest OsD14 binding activity was observed for GR24 (IC_50_: 1.24 ± 0.09 μM), followed by MP26 (IC_50_: 2.07 ± 0.80 μM), MP13 (IC_50_: 3.45 ± 1.2 μM), and finally AR8 (IC_50_ of 4.87 ± 0.78 μM). Docking of AR8 and MP13 to ShHTL7 active site further showed that the extra methyl group in AR8 could cause steric obstruction with the ShHTL7 Ile193 side chain, making it less active (Figure 7A). The de-methylated MP13 could bind to ShHTL7 without any steric hindrance, resulting in an increased affinity and more potent biological activities compared to AR8 (Figures S3A,B). Similarly, our docking analysis showed that OsD14 remained unaffected by the additional methyl group in AR8 since the binding residues are found well away without clashes, and our YLG assay showed that the affinity of OsD14 towards MP13 and AR8 is almost similar (Figure 7B; Figures S4A,B).

Testing of the Suicidal Germination Activity of Formulated SL Analogs on *Striga* Incidence Under Greenhouse and Naturally Infested Farmer Field Conditions.

**Figure 8:**
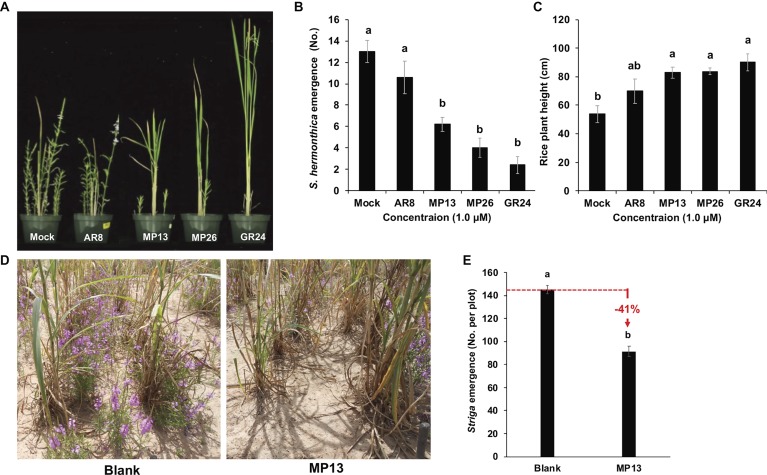
**(A)** *Striga hermonthica* emergence in pots under greenhouse conditions. **(B)** Number of *Striga* emergence per pot in response to SL analogs’ application. **(C)** Rice plant height in response to SL analogs’ application. About 4,000 *Striga hermonthica* seeds were thoroughly mixed in 2 L of sand and soil mixture. After 10 days of preconditioning, each pot was sprayed with 20 ml formulated SL analogs (conc. 1.0 μM) for five times up to 10 days. After 1 week of application, three rice seedlings were planted in the middle of each pot. Number of emerged *Striga* plants in each pot were counted after 8 weeks of sowing and plant height was measured at the time of final counting. We conducted this experiment two times and the same tendency was observed in both studies. **(D,E)** *Striga* emergence in response to suicidal effect of formulated MP13 application under naturally infested farmer field conditions. After onset of rainfall, formulated MP13 and normal water (control) were applied twice in each plot (25 ml m^−2^) following rains (≥ 10 mm) to get final concentration of 0.5 μM. The experiment was conducted in a randomized complete block design (*n* = 4). Bars represent number of *Striga* emergence per plot and compared by one-way ANOVA. Means not sharing a letter in common differ significantly at *p*_0.05_.

Next, we evaluated the *Striga* suicidal germination-inducing activity of MP13 and MP26 in artificially infested pots using rice as host crop (Figures 8A,B), by recording *Striga* emergence on weekly basis (Table S1). We observed highest *Striga* emergence in the AR8 treated pots (11 plants per pot) at 10 weeks after sowing (WAS), compared to other SL analogs tested. Nevertheless, AR8 treatment resulted in around 18% decrease in *Striga* emergence, compared to the mock control. At the same time point, pots treated with MP13 and MP26 showed an average of six and four *Striga* plants per pot, respectively, which corresponds to 52 and 69% reduction, compared to the mock treatment. Treatment with GR24 led to the most pronounced decrease (82%) in *Striga* emergence. Statistically, the effect of MP13 and MP26 was comparable to that of GR24 during the whole growth period. The severity of *Striga* infestation (number of emerging *Striga* plants) inversely correlated with the growth of the host (height). Plants grown in mock were the shortest one, followed by those grown in AR8-, MP13-, MP26-, and finally GR24-treated pots (Figure 8C).

We also assessed the activity of MP13 as suicidal germination agent in naturally *Striga*-infested farmer’s field in Burkina Faso, using our suicidal germination protocol developed for rain-fed agriculture of sub-Saharan Africa (Kountche *et al*., under review). For this purpose, we applied MP13 two times after onset of rainfall with final conc. of 0.5 μM. After few days of last application, a local pearl millet cultivar was sown as a host crop. At the end of the season, we counted 91 *Striga* plants m^−2^, compared to 154 plants m^−2^ in the mock field. In other words, application of formulated MP13 led to around 41% reduction in *Striga* emergence (Figures 8D,E).

## Discussion

The use of natural SLs to investigate their biological function and to understand how they act is less feasible because of their scarce and extremely low availability (10^−7^–10^−15^ M) from plant sources and the difficulties in producing them by organic synthesis (Joel, 2000; López-Ráez et al., 2009). Therefore, developing simple, efficient, and low-cost SL analogs remains an important task due to the importance of SLs for biologists and their potential for application in regulating crops’ architecture or as suicidal germination agents in combating root parasitic weeds, such as *S. hermonthica*. Many compounds can have SL activity if they only contain the D-ring (Cohen et al., 2013; Zwanenburg and Pospisil, 2013; Boyer et al., 2014). This information can be applied to develop some simple and low-cost SL analogs and mimics. Structural modification of SL analogs can have large impact on their efficiency. For example, the AR series, including AR8 and AR36, developed by Boyer et al. (2014), contain an additional methyl group on their D-ring (at the C-3′ atom) that is coupled to an acyclic carbon chain. The AR series did not require laborious synthesis. However, the activity of the AR analogs was relatively low, particularly with respect to the germination of root parasitic weeds. We set out to investigate the effect of this D-ring modification on the activity of AR8 and AR36 and to find out whether the removal of the methyl group at C-3′ atom can increase the activity of these analogs.

Since SLs are well known as plant growth regulators (Gomez-Roldan et al., 2008; Ruyter-Spira et al., 2011; Brewer et al., 2013; Al-Babili and Bouwmeester, 2015), we first examined some growth-regulating activities of MP13 and MP26 in rice and *Arabidopsis*. In one study, we applied SL analogs at 2.5 μM in a hydroponic culture system and compared them with GR24 and AR8 to see wild type rescuing phenotype on high-tillering SL-deficient rice *d10/CCD8* mutant (Figures 3A,B). MP13 and MP26 reduced tillering in *d10* mutant as compared with mock treatment. This effect was significantly higher than AR8 but less than GR24. We observed similar effect of MP26 on hypocotyl length and lateral root density in *Arabidopsis*, showing considerable inhibition than AR8 but less than GR24 (Figures S1A,B). Although *rac*-GR24 inhibits hypocotyl growth through both D14 and KAI2, it can be assumed that KAI2 is the main hypocotyl growth regulator, as suggested by hypocotyl length phenotype of the corresponding loss of function mutants (Waters et al., 2012b). Thus, the weak activity of MP26 and the lack of MP13 effect on hypocotyl length suggest that these SL analogs likely transduce their signal through D14 rather than KAI2. Contrary to our previously described nitro-phenlactone (Jia et al., 2016), the newly developed MP26 has shown reasonable activity in regulating lateral root density. These improved activities indicate the possibility to employ MP13 and MP26 as simple and cheaper growth regulators in the future. The synthesis of GR24 requires much more steps (Zwanenburg et al., 2016c) than that of MP13 and MP26. Hence, it can be assumed that the production of our two SL analogs will cost much less than that of GR24.

We are very interested in developing simple and low-cost SL analogs to combat root parasitic weeds, in particular *Striga* that severely threats food security in sub-Saharan Africa. Such SL analogs can be applied in the absence of a host, which would lead to the death of *Striga* seedlings at very early stage, a strategy called suicidal germination. Therefore, we first tested the seed germination-inducing activity of MP13 and MP26 under lab conditions, in comparison to *rac*-GR24 and AR8. The two MP analogs were much more active in inducing seed germination in *Striga* and *Phelipanche* than AR8, which was confirmed by calculating the corresponding EC_50_ values. Interestingly, both MP13 and MP26 (1.0 μM concentration) showed high activity with *P. aegyptiaca*, which was statistically similar to that of GR24.

Stability is a major factor in determining the activity of SL analogs. Therefore, we determined the stability of MP13 and MP26 in formulated and non-formulated forms, and under different conditions, including incubation in sand at 30°C. In general, our results show that GR24 is the most stable analog tested in this study, followed by MP26, MP13, and finally AR8. Stability test showed that MP13 and MP26 are less stable than GR24 in sand. This result indicates that our new analogs might have less impact on the environment, compared to GR24. Nevertheless, toxicity tests and precise evaluation of the effect on soil organisms need to be provided before applying these compounds in field. It is important to note that although MP13 showed early degradation in HPLC, it still showed better *Striga* germination-inducing activity than AR8. We hypothesized that the observed increase in *Striga* germination activity by modified MPs is due to their high affinity to *Striga* receptor. We confirmed this hypothesis by assessing the competitive inhibition of ShHT7 and OsD14-mediated YLG hydrolysis assays. In these experiments, the lower IC_50_ value (and hence stronger ShHTL7 and OsD14 interaction) of MP13 and MP26 compared with AR8 explained the increased sensitivity of *Striga* and rice to MP13 and MP26. The higher activity of MP26 for *Striga* germination despite a 2-fold lower IC_50_ compared to MP13 might be due to the increased stability of MP26. While better tillering inhibition by MP26 than MP13 and AR8 might be due to its higher binding activity to OsD14 (Figures 6A,B). Our computational structural analysis suggested that the increased ShHTL7- and OsD14-binding affinity of MP13 compared to AR8 is explained by the additional methyl group in AR8 causing a steric hindrance with Ile193 of the ShHTL7 active site. Conversely, MP13 lacking that methyl group could bind to ShHTL7 and OsD14 without such clashes. Whereas OsD14 found to be less affected by the presence of additional methyl group in AR8 and the results are in accordance with the binding affinity toward MP13 and AR8 (Figures 7A,B).

To get information on the application potential of MP13 and MP26, we first tested their *Striga* germination-inducing activity in pots under greenhouse conditions. This test showed quite promising results. The application of formulated MP13 and MP26 significantly alleviated *Striga* emergence. The effect of these compounds was much higher than that of AR8, comparable with GR24 (Figures 8A–C). The inspiring pot results led us to test the activity of MP13 under real conditions in naturally infested African field in Burkina Faso. The reason for testing MP13 instead of the more active MP26 was the availability of the former at the time of application. In this field trial, we used our very recently developed protocol that demonstrated the practicability of the suicidal germination approach in rain-fed agriculture in sub-Saharan Africa (Kountche et al., under review). The application of MP13 in an infested pearl millet field led to 41% reduction in *Striga* infestation, compared with the corresponding control (Figures 8D,E). This activity is comparable to that of MP1, a methyl carlactonoate-based analog that we have very recently tested under the same conditions (Jamil et al., 2018). It should be mentioned here that the final concentration required for this activity is estimated to be around 0.5 μM, calculated based on 10-mm rainfall that distributes the analog in soil, which corresponds to 18 g a.i. ha^−1^. This amount is significantly less than the amounts used to apply the SL analog T-010 as suicidal germination agent (Samejima et al., 2016). It can be assumed that the application of MP26 may lead to even better results, given the better performance of this compound under lab and greenhouse conditions. A corresponding field test in Burkina Faso is planned for the future. Although, our results confirm the practicability of the suicidal germination strategy and unravel MP13 and MP16 as very promising suicidal germination agents for infested African fields, however, the application method still requires improvements, and further studies are needed to optimize the number, time, and dose of applications.

Our findings show that the presence of methyl group at the C-3′ in D-ring has a negative impact on SL analogs’ activity and provides structural explanation for this effect. The developed MP13 and MP26 are promising SL analogs that can be applied to regulate crop architecture. MP13 has large potential for application in combating the root parasitic weed *Striga* in infested regions of sub-Saharan Africa.

## Author Contributions

SA-B and MJ conceived and designed the experiments. BK, IH, JW, FA, RZ, K-PJ, DY, IT, TO, and TA performed the experiments. UH and SA did the *in silico* analysis. MJ, SA, TA, SA-B, and others wrote the manuscript and respective parts. All authors read, edited, and approved the final manuscript.

### Conflict of Interest Statement

The authors declare that the research was conducted in the absence of any commercial or financial relationships that could be construed as a potential conflict of interest.

## Footnotes

^1^http://rsbweb.nih.gov/ij/

^2^http://www.ic50.tk/index.html

## Supplementary Material

FIGURE S1 Effect of SL analogs on rice growth. The SL analogs were applied (2.5 μM) to 1-week old hydroponically grown rice seedlings (*d10* mutant) twice a week up to 3 weeks. Plant height and fresh biomass per plant were recorded (*n* = 8) and compared by one-way ANOVA. Means not sharing a letter in common differ significantly at *p*_0.05_.

FIGURE S2 Effect of SL analogs on *Arabidopsis* hypocotyl length **(A)**. Number of lateral roots per plant **(B)**. Eight days old *Arabidopsis* seedlings (at least 30) treated with each compound were photographed digitally and, hypocotyl length and lateral root density were measured using ImageJ software. Bars represent means ± SE. Means not sharing a letter in common differ significantly at *p*_0.05_.

FIGURE S3 Ligplot showing the residues of ShHTL7 forming close contacts with AR8 **(A)** and MP13 **(B)**, respectively.

FIGURE S4 Ligplot showing the residues of OsD14 forming close contacts with AR8 **(A)** and MP13 **(B)**, respectively.

TABLE S1 Weekly *Striga hermonthica* emergence in response to suicidal effect of formulated strigolactone analogs application in pots under green-house conditions.

TABLE S2 Physico-chemical properties of MP13 and MP26.
