# Supplementary material for: Methylation at the C-3′ in D-Ring of Strigolactone Analogs Reduces Biological Activity in Root Parasitic Plants and Rice
Source: Front Plant Sci. 2019 Apr 2;10:353. doi: 10.3389/fpls.2019.00353 (PMC6455008; doi:10.3389/fpls.2019.00353)
Supplement: FIGURE S1 — Effect of SL analogs on rice growth. The SL analogs were applied (2.5 μM) to 1-week old hydroponically grown rice seedlings (d10 mutant) twice a week up to 3 weeks. Plant height and fresh biomass per plant were recorded (n = 8) and compared by one-way ANOVA. Means not sharing a letter in common differ significantly at p0.05. [file Data_Sheet_1.PDF]

Figure S1

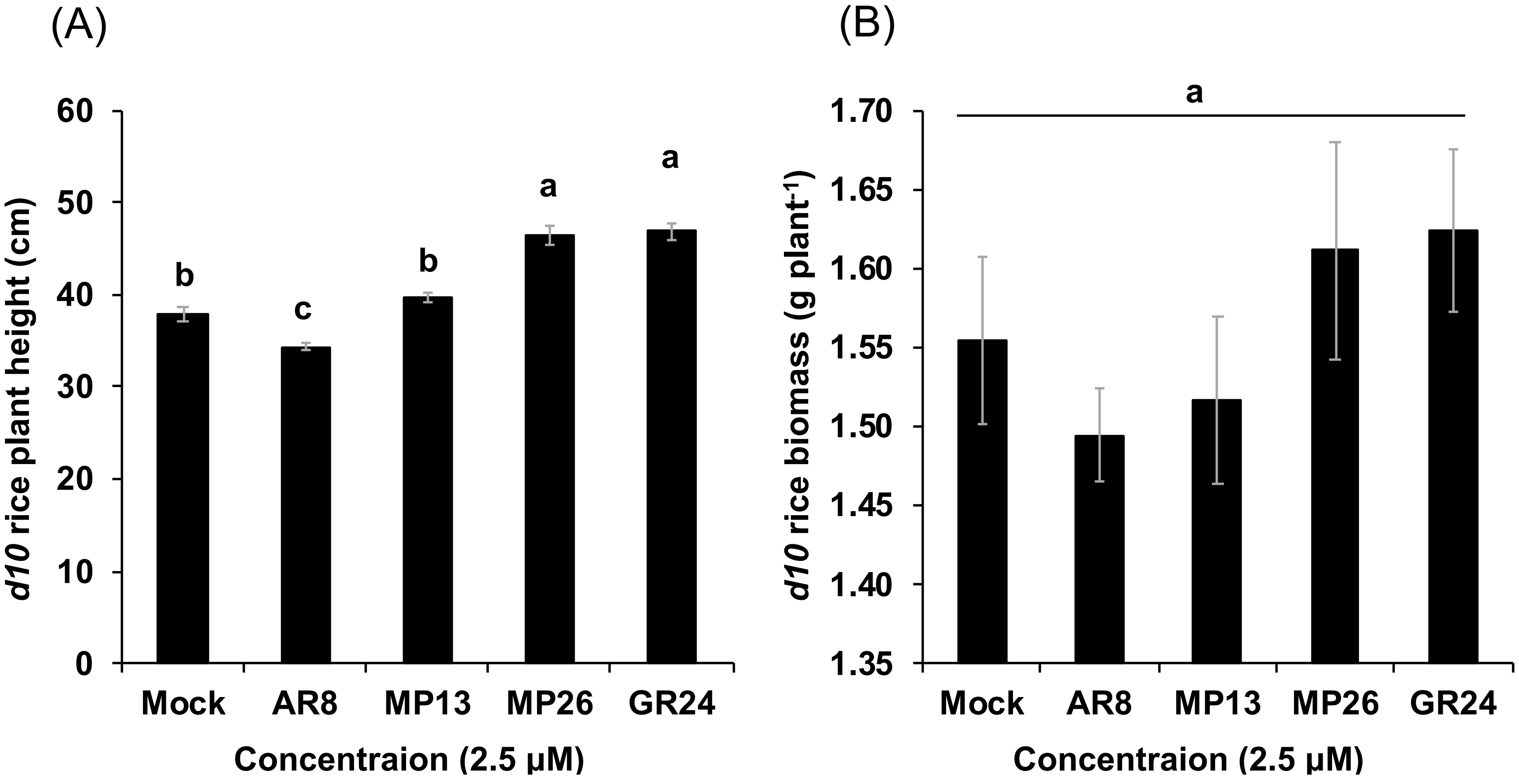
(A)
60
50
40
30
20
10
0
d10 rice plant height (cm)
b
c
b
a
a
Mock
AR8
MP13
MP26
GR24
Concentraion (2.5 µM)
(B)
a
1.70
1.65
1.60
1.55
1.50
1.45
1.40
1.35
d10 rice biomass (g plant-1)
Mock
AR8
MP13
MP26
GR24
Concentraion (2.5 µM)

Figure S2

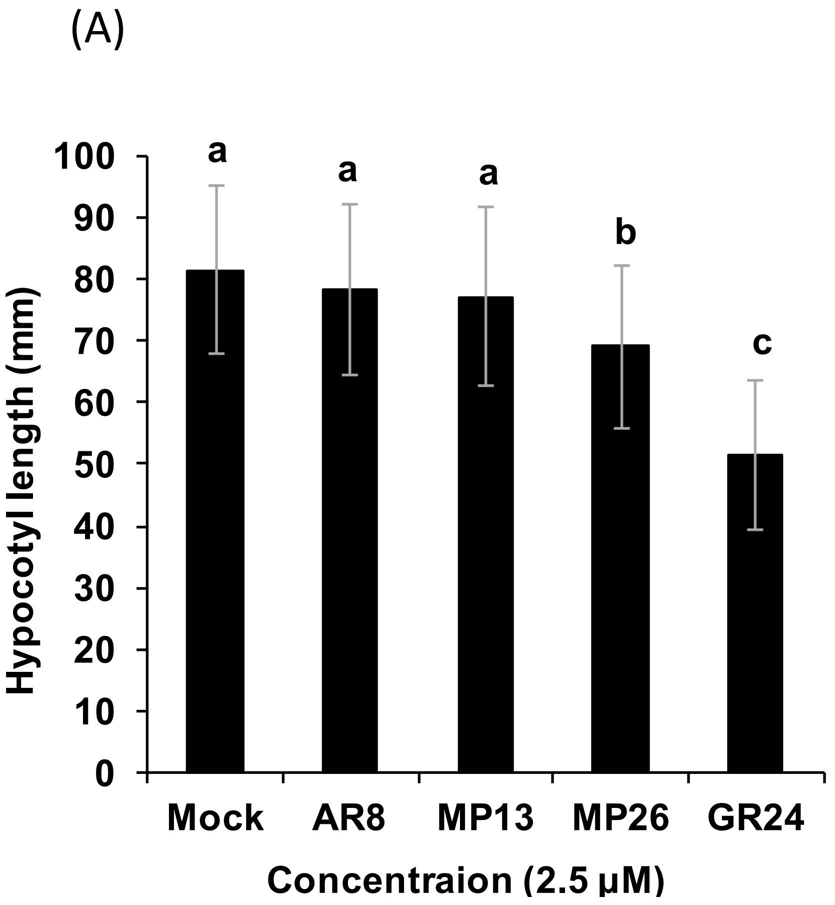

(A)
a
a
a
b
c
100
90
80
70
60
50
40
30
20
10
0
Hypocotyl length (mm)
Mock
AR8
MP13
MP26
GR24
Concentraion (2.5 μM)

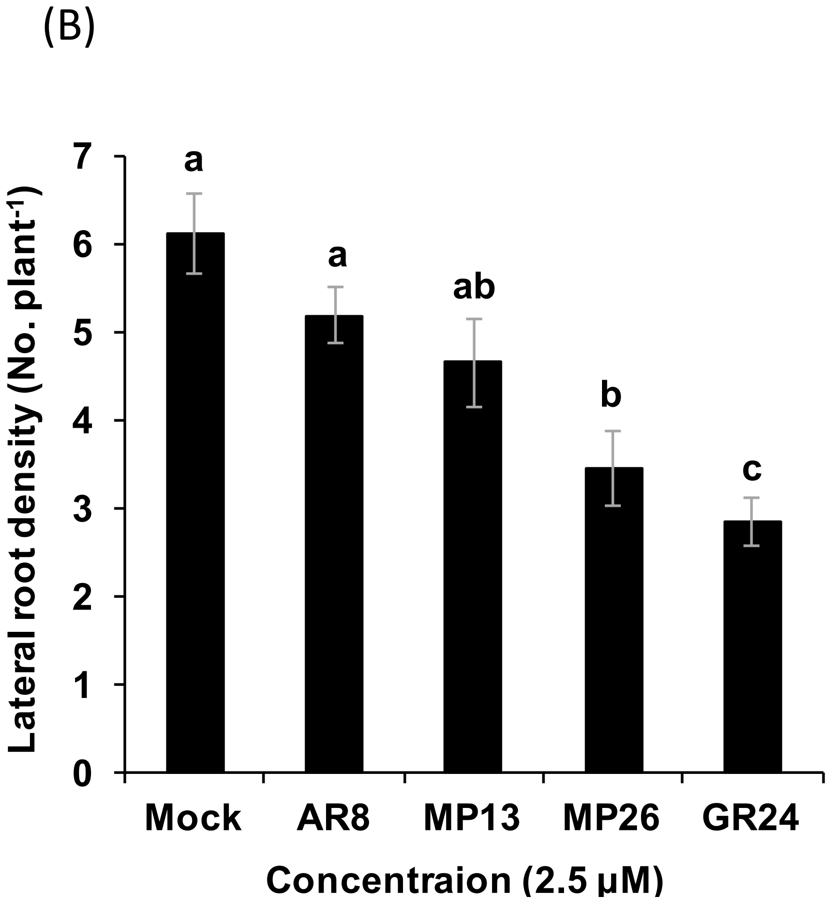

(B)
a
a
ab
b
c
7
6
5
4
3
2
1
0
Lateral root density (No. plant-1)
Mock
AR8
MP13
MP26
GR24
Concentraion (2.5 μM)

Figure S3

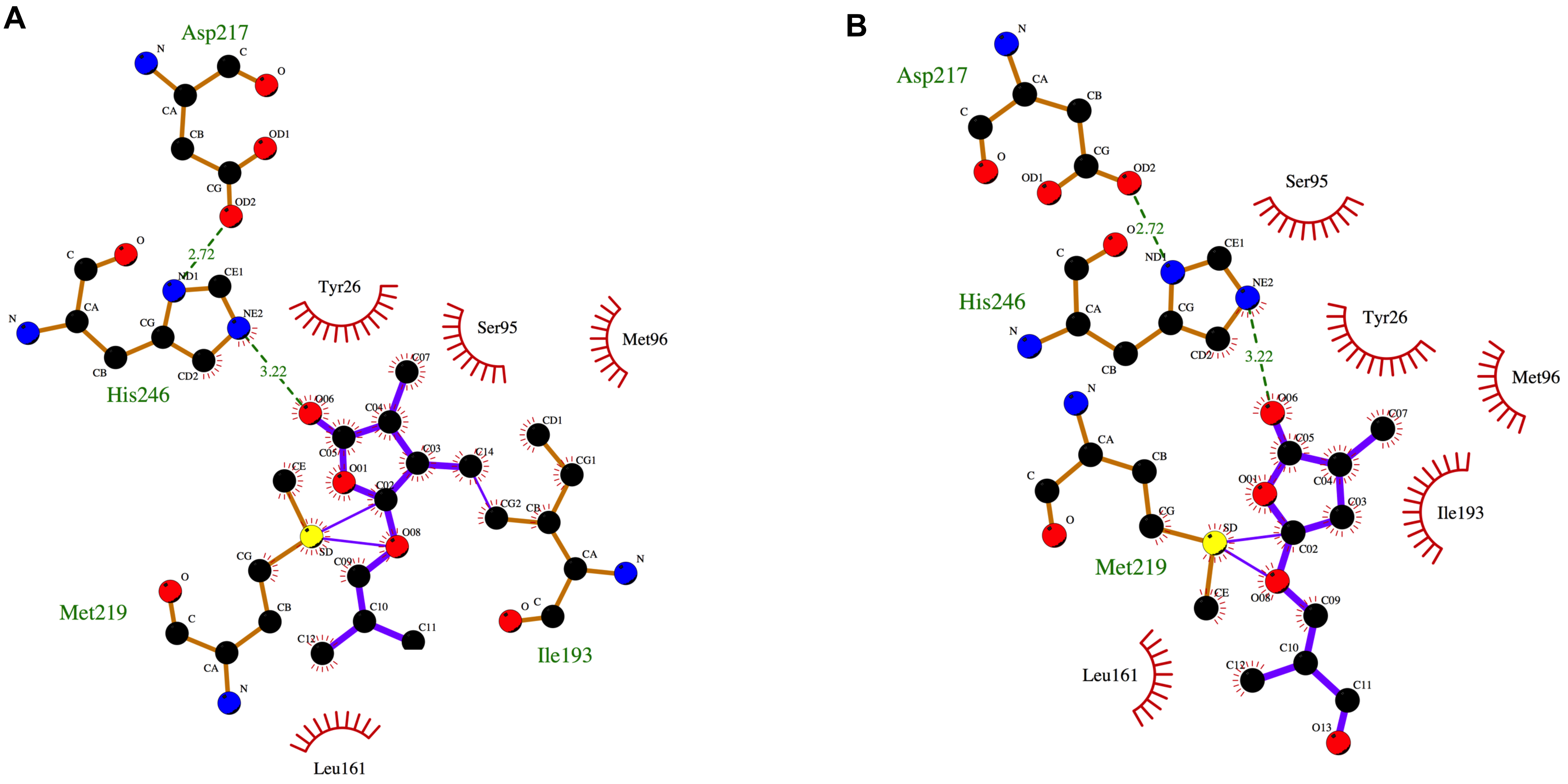

AR8

MP13

Figure S4

A

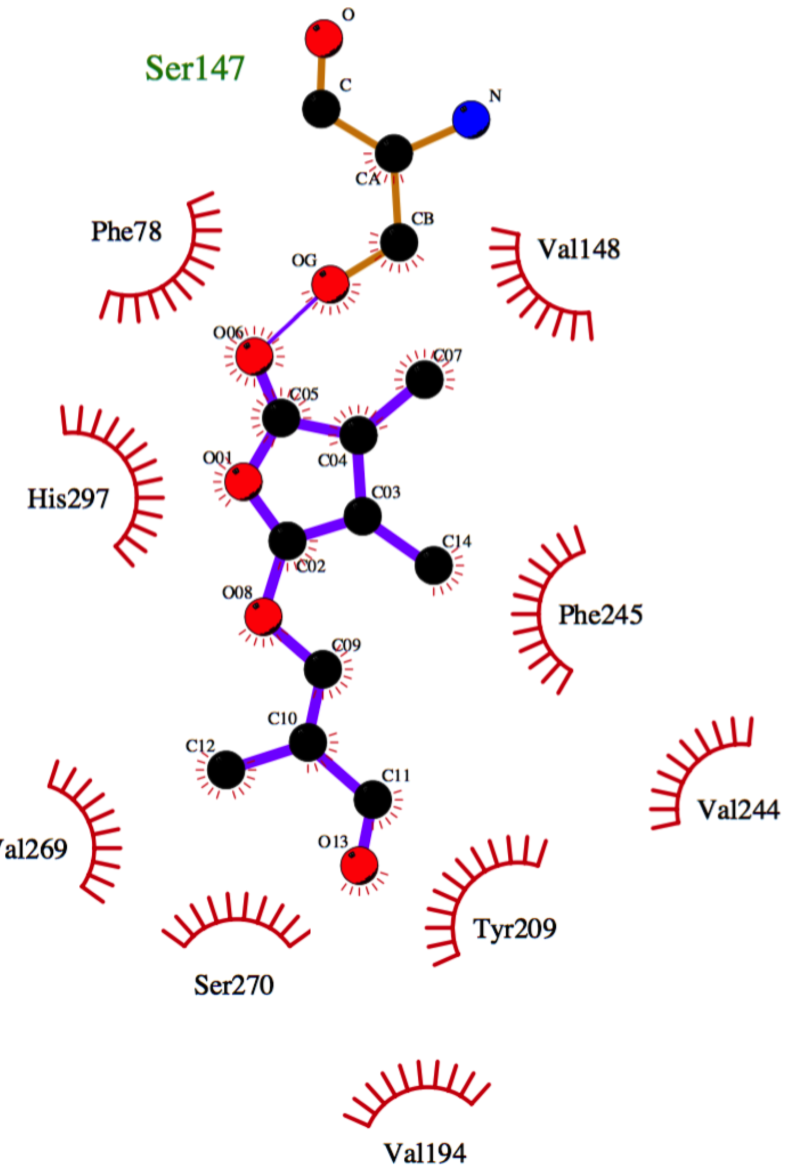

AR8

B

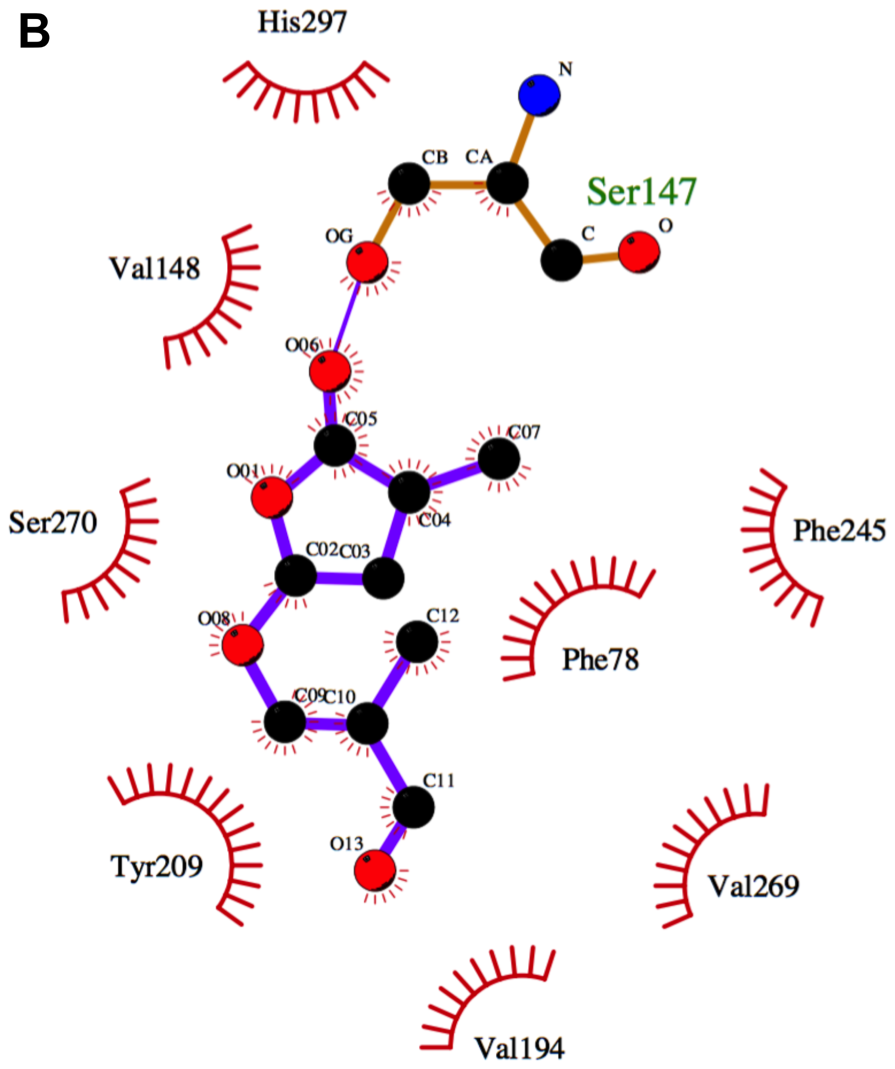

MP13

Table S1 Weekly *Striga hermonthica* emergence in response to formulated strigolactone analogs application in pots under green house conditions

**Treatments**	**5 WAS**	**6 WAS**	**7 WAS**	**8 WAS**	**9 WAS**	**10 WAS**
Mock	2.2±0.4a[1] (0)[2]	4.8±0.7a (0)	8.0±1.3a (0)	11.0±1.0a (0)	12.2±1.0a (0)	13.0±1.0a (0)
AR8	0.4±0.2b (-8)	4.4±0.5a (-10)	7.2±1.5a (-4)	10.6±1.5a (-4)	10.8±1.5a (-11)	10.6±1.5a (-18)
MP13	0.0±0.0b (-67)	1.6±1.0b (-68)	2.6±0.6b (-56)	4.8±0.6b (-56)	5.8±0.6b (-51)	6.2±0.7b (-52)
MP26	0.0±0.0b (-83)	0.8±0.6b (-83)	1.4±0.4b (-76)	2.6±0.4b (-76)	3.6±0.8bc (-68)	4.0±0.9b (-69)
GR24	0.0±0.0b (-100)	0.0±0.0b (-88)	1.0±0.6b (-85)	1.6±0.5b (-85)	1.6±0.5c (-84)	2.4±0.8b (-82)

WAS week after sowing
1. Values represent means ± SE (*n*=5); Means not sharing a letter in common are differ significantly at $P_{0.05}$.
2. Negative values in parenthesis show percent decrease in *Striga hermonthica* emergence over mock

Table S2

	**Physico-Chemical Properties**
MP13	^{1}H NMR (500MHz, $CDCl_3$): δ 9.34 (1H, s), 7.17 (1H,s), 6.98 (1H, s), 6.19 (1H, s), 2.04 (3H, s), 1.70 (3H, s). HRMS-Esi: m/z $[M\text{-}Na]^-$ Calcd for $C_9H_{10}Na_1O_4$:205.04768, found: 205.06819. 13C-NMR (126 MHz, CDCl3) δ: 192.19, 170.54, 162.58, 141.40, 135.18, 122.52, 100.29, 10.38, 6.20. Melting point 88℃.
MP26	^{1}H NMR (500MHz, $CDCl_3$): δ 7.25 (1H, d, *J* = 15 Hz), 6.91 (1H, s), 6.73 (1H, s), 6.06 (1H, s), 5.81(1H, d, *J* = 15 Hz), 3.74 (3H, s), 2.00 (3H, s), 1.74 (3H, s). 13C-NMR (126 MHz, CDCl3) δ: 170.70, 167.66, 148.56, 144.82, 141.55, 135.22, 117.16, 115.06, 100.00, 51.39, 10.62, 9.31. Melting point 146-149℃.
